# Strongly exchange-coupled and surface-state-modulated magnetization dynamics in Bi_2_Se_3_/yttrium iron garnet heterostructures

**DOI:** 10.1038/s41467-017-02743-2

**Published:** 2018-01-15

**Authors:** Y. T. Fanchiang, K. H. M. Chen, C. C. Tseng, C. C. Chen, C. K. Cheng, S. R. Yang, C. N. Wu, S. F. Lee, M. Hong, J. Kwo

**Affiliations:** 1 Department of Physics, National Taiwan University, Taipei, 10617 Taiwan; 2 Department of Physics, National Tsing Hua University, Hsinchu, 30013 Taiwan; 3 Institute of Physics, Academia Sinica, Taipei, 11529 Taiwan

## Abstract

Harnessing the spin–momentum locking of topological surface states in conjunction with magnetic materials is the first step to realize novel topological insulator-based devices. Here, we report strong interfacial coupling in Bi_2_Se_3_/yttrium iron garnet (YIG) bilayers manifested as large interfacial in-plane magnetic anisotropy (IMA) and enhancement of damping probed by ferromagnetic resonance. The interfacial IMA and damping enhancement reaches a maximum when the Bi_2_Se_3_ film approaches its two-dimensional limit, indicating that topological surface states play an important role in the magnetization dynamics of YIG. Temperature-dependent ferromagnetic resonance of Bi_2_Se_3_/YIG reveals signatures of the magnetic proximity effect of *T*_C_ as high as 180 K, an emerging low-temperature perpendicular magnetic anisotropy competing the high-temperature IMA, and an increasing exchange effective field of YIG steadily increasing toward low temperature. Our study sheds light on the effects of topological insulators on magnetization dynamics, essential for the development of topological insulator-based spintronic devices.

## Abstract (web summary)

Understanding the effects of topological insulators on magnetization dynamics of adjacent magnetic materials is essential for novel spintronic devices. Here, Fanchiang et al. report thickness dependence of interfacial in-plane magnetic anisotropy and damping enhancement in Bi_2_Se_3_/yttrium iron garnet (YIG) bilayers, indicating an important role of topological surface states in the magnetization dynamics of YIG.

## Introduction

The development of spintronics relies crucially on control of spin-polarized currents, which carry spin angular momenta that can be utilized to manipulate magnetic moments through spin-transfer processes. Spin currents can be generated by the spin Hall effect^1^ in a heavy metal, or by exploiting the spin structure of some two-dimensional (2D) electron systems. A promising candidate of such a 2D system is the surface state of topological insulators (TIs). TIs are emergent quantum materials hosting topologically protected surface states, with dissipationless transport prohibiting backscattering^2,3^. Strong spin–orbit coupling (SOC) along with time reversal symmetry (TRS) ensures that the electrons in the topological surface states (TSSs) have their direction of motion and spin locked to each other^2,4,5^. The spin–momentum locking permits efficient interconversion between spin and charge currents. To date, several methods have been adopted to estimate the spin-charge conversion efficiency of TIs, either by using microwave-excited dynamical method^6–10^ (e.g., spin pumping and spin–torque ferromagnetic resonance (ST-FMR)) or thermally induced spin injection^11^. Very large values of spin-charge conversion ratio have been reported^7,9,10^. Recently, TIs are shown to be excellent sources of spin–orbit torques (SOT) for efficient magnetization switching^12^.

When a TI is interfaced with a magnetic layer, the interfacial exchange coupling can induce magnetic order in TIs by the magnetic proximity effect (MPE) and break the TRS^13–16^. The resulting gap opening of the Dirac state is necessary to realize novel phenomena such as topological magneto-electric effect^17^ and quantum anomalous Hall effect^18,19^. Since the MPE and spin-transfer process rely on interfacial exchange coupling of TI/ferromagnet, understanding the magnetism at the interface has attracted strong interests in recent years. Several techniques have been adopted to investigate the interfacial static magnetic properties, including spin-polarized neutron reflectivity^15,20^, second harmonic generation^21^, electrical transport^14,22^, and magneto-optical Kerr effect^14^. All these studies clearly indicate the existence of MPE resulting from exchange coupling and strong SOC in TIs. Specifically, a room-temperature magnetic order induced by MPE in EuS/Bi_2_Se_3_ has been reported recently^15^. Through exchange coupling between the TSS and EuS layer, the induced magnetic moments exhibited perpendicular magnetic anisotropy (PMA) that can potentially open a gap of TSS. For TI/yttrium iron garnet (YIG) bilayer, however, the interfacial magnetic anisotropy and the resulting magnetization dynamics under the influence of TSS are still largely unknown. It is equally important to understand how the interfacial exchange coupling affects the magnetization dynamics of Bi_2_Se_3_/YIG because of the wide applications of YIG. For example, TIs can enhance the magnetic anisotropy, introduce additional magnetic damping, and greatly alter the dynamical properties of the ferromagnetic layer, as commonly observed in ferromagnet/heavy metals systems^23–25^. The enhanced damping is visualized as larger linewidth of FMR spectra^23–25^. Given the volatile surface band structure depending on the TI thickness^26^, the adjacent materials^27^, and the magnetism at the interfaces^28^, experimental study on how the magnetization dynamically responds to the TSS is still lacking, which is a topic not only important for spintronics but also fundamental for physics.

In this work, we have investigated the magnetization dynamics via FMR in ferrimagnetic insulator YIG under the influence of the prototypical three-dimensional (3D) TI Bi_2_Se_3_^29^. We choose YIG as the ferromagnetic layer because of its technological importance, with high *T*_C_ ~550 K and extremely low damping coefficient *α*^30^. When YIG is interfaced with TIs, its good thermal stability minimizes the interdiffusion of materials. Through the Bi_2_Se_3_ thickness dependence study, we observed a strong modulation of FMR properties attributed to the TSS of Bi_2_Se_3_. The temperature-dependent study unraveled an effective field parallel to the magnetization direction existing in Bi_2_Se_3_/YIG. Such an effective field built up as the temperature decreased, which was utilized to demonstrate the zero-applied-field FMR of YIG. Furthermore, we identified a possible signature of MPE of *T*_C_ as high as 180 K manifested as enhanced spin pumping in a fluctuating spin system, as well as a small emerging PMA at low temperature in competition with in-plane magnetic anisotropy (IMA) extending to high temperature.

## Results

### Interfacial IMA in Bi_2_Se_3_/YIG

**Fig. 1:**
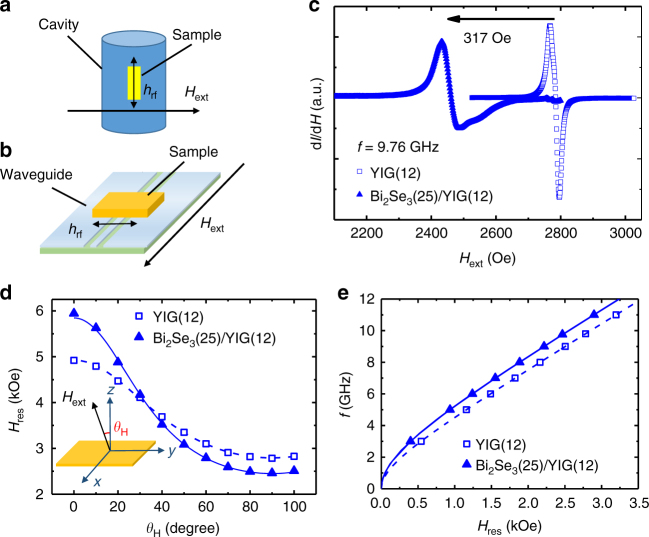
Schematic diagrams and results of the angle- and frequency-dependent FMR measurements. **a**, **b** FMR using the cavity and co-planar waveguide configuration for angle- and frequency-dependent study, respectively. A dc external field *H*_ext_ was applied and *h*_rf_ denotes the microwave field. **c** FMR spectra of Bi_2_Se_3_(25)/YIG(12) and YIG(12) measured by the cavity. **d**, **e** *θ*_H_ and *f* dependence of *H*_res_ of Bi_2_Se_3_(25)/YIG(12) and YIG(12), respectively

The room-temperature FMR measurements were performed using a microwave cavity of frequency 9.76 GHz (Fig. 1a) and a broadband coplanar waveguide (Fig. 1b). The FMR spectra in Fig. 1c are compared for single layer YIG(12) and Bi_2_Se_3_(25)/YIG(12) bilayer (digits denote thickness in nanometer), showing a large shift of resonance field (*H*_res_) ~317 Oe after the Bi_2_Se_3_ growth plus a markedly broadened peak-to-peak width Δ*H* for Bi_2_Se_3_/YIG. Figure 1d shows *H*_res_ vs. applied field angle with respect to the surface normal *θ*_H_ for YIG(12) and Bi_2_Se_3_(25)/YIG(12). Larger variation of *H*_res_ with *θ*_H_ in the bilayer sample was observed. When the applied field was directed in the film plane, clear negative *H*_res_ shifts induced by Bi_2_Se_3_ were observed at all microwave frequencies *f* as shown in Fig. 1e. The data in Fig. 1d, e can be fitted in the scheme of magnetic thin films having uniaxial PMA, the strength of which is characterized by the effective demagnetization field 4*πM*_eff_ = 4*πM*_s_ − *H*_an_ − *H*_int_, where 4*πM*_s_, *H*_an_, and *H*_int_ are the demagnetization field, the magnetocrystalline anisotropy field of YIG, and the interfacial anisotropy field induced by Bi_2_Se_3_, respectively. The fitting result shows an ~60% enhancement of 4*πM*_eff_ for the Bi_2_Se_3_(25)/YIG(12) bilayer sample. The large enhancement cannot be accounted for by an increase in the saturation magnetization *M*_s_, which should amount to an additional magnetization of ~100 *μ*_B_/nm^2^ for this sample. The MPE, even if it persists up to room temperature, is unlikely to induce the large amounts of magnetic moments. Furthermore, since the x-ray diffraction results in Supplementary Fig. 3(d) and (e) show that the YIG films did not gain additional strain after growing Bi_2_Se_3_, the enhanced anisotropy cannot result from the change of magnetocrystalline anisotropy. We thus attribute the change of anisotropy mostly to the *H*_int_. Based on the above discussion, we obtain *H*_int_ = −926 and −1005 Oe from Fig. 1d, e, respectively (see Supplementary Note 2). The minus sign indicates the additional anisotropy points in the film plane.

**Fig. 2:**
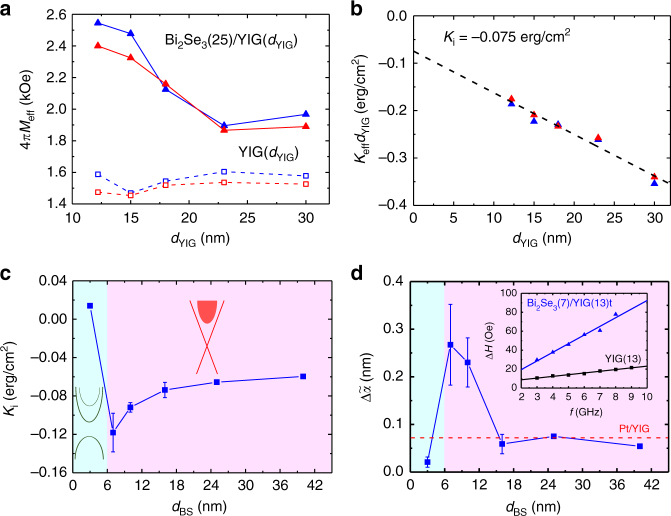
YIG and Bi_2_Se_3_ thickness dependence of FMR characteristics of Bi_2_Se_3_/YIG. **a** The *d*_YIG_ dependence of 4*πM*_eff_ of Bi_2_Se_3_/YIG (solid triangles) and YIG (hollow squares) obtained from *θ*_H_ (red) and *f* (blue) dependent FMR. **b** The *K*_eff_*d*_YIG_ vs. *d*_YIG_ plot for determining *K*_i_ using a linear fit. The intercept of the *y*-axis corresponds to the *K*_i_ value. **c** *d*_BS_ dependence of *K*_i_. The figure is divided into two regions. For *d*_BS_ >6 nm, the Dirac cone of TSS is intact, with the Fermi level located in the bulk conduction band. For *d*_BS_ <6 nm, a gap and quantum well states form. **d** The *d*_BS_ dependence of normalized damping enhancement $\Delta \tilde \alpha$. The inset shows Δ*H* as a function of *f* Bi_2_Se_3_(7)/YIG(13) and YIG(13) for calculating *α*_BS/YIG_ − *α*_YIG_. The red dashed line shows the typical value of $\Delta \tilde \alpha$ of Pt/YIG for comparison. The error bars indicate standard deviations of at least four samples

The above observations suggested the presence of interfacial IMA in Bi_2_Se_3_/YIG. To verify this, we systematically varied the thickness of YIG, *d*_YIG_, while fixing the thickness of Bi_2_Se_3_. Figure 2a presents the *d*_YIG_ dependence of 4*πM*_eff_ for single and bilayer samples. The 4*πM*_eff_ of single layer YIG was independent of *d*_YIG_ varying from 12 to 30 nm. In sharp contrast, 4*πM*_eff_ of Bi_2_Se_3_/YIG became significantly larger, especially at thinner YIG, which is a feature of an interfacial effect. The *f*- and *θ*_H_-dependent FMR were performed independently to doubly confirm the trends. The interfacial IMA can be further characterized by defining the effective anisotropy constant *K*_eff_ = (1/2)4*πM*_eff_*M*_s_ = (1/2)(4*πM*_s_ − *H*_an_)*M*_s_ − *K*_i_/*d*_YIG_, with the interfacial anisotropy constant *K*_i_ = *M*_s_*H*_int_*d*_YIG_/2. The *K*_eff_*d*_YIG_ vs. *d*_YIG_ data in Fig. 2b are well fitted by a linear function, indicating that the *d*_YIG_ dependence presented in Fig. 2a is suitably described by the current form of *K*_eff_. The intercept obtained by extrapolating the linear function corresponds to *K*_i_ = −0.075 erg/cm^2^.

### TSS-modulated magnetization dynamics in Bi_2_Se_3_/YIG

To further investigate the physical origin of the IMA, we next varied the thickness of Bi_2_Se_3_ (*d*_BS_) to see how *K*_i_ evolved with *d*_BS_. Figure 2c shows the *d*_BS_ dependence of *K*_i_. Starting from the *d*_BS_ = 40 nm sample, the magnitude of *K*_i_ went up as *d*_BS_ decreased. An extremum of *K*_i_ −0.12 ± 0.02 erg/cm^−^^2^ was reached at *d*_BS_ = 7 nm. An abrupt upturn of *K*_i_ occurred in the region 3 nm < *d*_BS_ < 7 nm. The *K*_i_ magnitude dropped drastically and exhibited a sign change in the interval. Furthermore, the *K*_i_ value of 0.014 erg/cm^2^ at *d*_BS_ = 3 nm corresponds to weak interfacial PMA.

The sizable interfacial IMA can be expected given the large SOC of Bi_2_Se_3_. One possible mechanism is that the electrons at the interface re-distribute upon hybridization between the Fe *d*-orbital of YIG and the Dirac surface state of Bi_2_Se_3_. Recent theoretical study on EuS/Bi_2_Se_3_ bilayers indicates that in addition to the strong SOC, TSS play a crucial role in mediating the exchange coupling of the ions in the magnetic layer^31^. The hybridization between TSS and the magnetic layer can overall enhance the magnetic anisotropy energy that is inherent at the interface^31^. Although in general an interfacial magnetic anisotropy may not necessarily be related to the topological nature of materials, here we attribute the interfacial magnetic anisotropy of Bi_2_Se_3_/YIG to the TSS based on the unique *d*_BS_ dependence of *K*_i_, that cannot be accounted for by the strain or chemical mixing effects. Note that possible interdiffusion of materials at the interface can also lead to an interfacial magnetic anisotropy. As shown in Supplementary Fig. 3(c), the transmission electron microscope (TEM) image reveals an ~1 nm interfacial layer. However, the interdiffusion is unlikely to play a dominant role in the interfacial magnetic anisotropy since *K*_i_ varied significantly with *d*_BS_ up to 40 nm, and cannot account for the modulated dependence of *K*_i_ with *d*_BS_, especially under 20 nm. We now consider how the Bi_2_Se_3_ band structure evolves with *d*_BS_. Based on previous investigation on surface band structure of ultrathin Bi_2_Se_3_^26^, *d*_BS_ = 6 nm was identified as the 2D quantum tunneling limit of Bi_2_Se_3_. When *d*_BS_ < 6 nm, the hybridization of top and bottom TSS developed a gap in the surface states. Spin-resolved photoemission study later showed that the TSS in this 2D regime exhibited decreased in-plane spin polarization^32^. The modulated spin texture may lead to the weaker interfacial magnetic anisotropy than that in the 3D regime^32,33^. We thus divide Fig. 2c into two regions and correlate the systematic magnetic properties with the surface state band structure. The sharp change of *K*_i_ around *d*_BS_ < 6 nm strongly suggests that the interfacial IMA in Bi_2_Se_3_/YIG is of topological origin.

The Δ*H* broadening in FMR spectra after growing Bi_2_Se_3_ on YIG indicates that Bi_2_Se_3_ introduced additional damping in YIG. Within the macrospin approximation, the damping enhancement can be normalized with respect to *d*_YIG_ by defining $\Delta \tilde \alpha ={d_{{\mathrm Y} {\mathrm I} {\mathrm G}} \left( \alpha_{{\mathrm B} {\mathrm S}/ {\mathrm Y} {\mathrm I} {\mathrm G}}-\alpha_{{\mathrm Y} {\mathrm I} {\mathrm G}} \right)}$, where *α*_BS/YIG_ and *α*_YIG_ are the damping coefficient of Bi_2_Se_3_/YIG and YIG, respectively. Figure 2d displays the *d*_BS_ dependence of $\Delta \tilde \alpha$. Similar to *K*_i_ in Fig. 2c, $\Delta \tilde \alpha$ increased as *d*_BS_ decreased, reached its maximum at *d*_BS_ = 7 nm with a very large value of ~0.27 nm, and then dropped abruptly in the interval of 3 nm < *d*_BS_ < 7 nm. For comparison, typical $\Delta \tilde \alpha$ of Pt/YIG, in which efficient spin pumping giving rise to sizable $\Delta \tilde \alpha$^24^, is indicated by the red dashed line. The inset shows Δ*H* vs. *f* data for Bi_2_Se_3_ (7)/YIG(13) and YIG(13) fitted by linear functions. One can clearly see a significant change of slope, from which we determined *α*_BS/YIG_ − *α*_YIG_ to be 0.014. In general, the large damping enhancement can have multiple origins, including spin-pumping effect, interlayer exchange coupling with other magnetic layers, and chemical reactions at the interface. However, the damping arising from the static exchange coupling from the MPE or any antiferromagnetic order at the interface is not expected at room temperature. Moreover, as previously mentioned, the slight interdiffusion at the interface is unlikely to be the major root cause of $\Delta \tilde \alpha$ varying over the wide range of *d*_BS_. Instead, considering the closed *d*_BS_ dependence of *K*_i_ and $\Delta \tilde \alpha$, it can be seen that the trend of $\Delta \tilde \alpha$ in Fig. 2d stemmed from the strong coupling between TSS of Bi_2_Se_3_ and YIG—that is, the surface state band structure of Bi_2_Se_3_ profoundly affected the damping of YIG^6^. Through dynamical exchange, spin angular momenta were transferred from YIG to the TSS via the spin-pumping effect. The spin-pumping efficiency of an interface can be evaluated by the real part of spin mixing conductance *g*_↑↓_ using the following relation^25^:

1 $$g_{ \uparrow \downarrow } = \frac{{4\pi M_{\mathrm{s}}d_{{\mathrm{YIG}}}}}{{g\mu _{\mathrm{B}}}}\left( {\alpha _{{\mathrm{BS}}/{\mathrm{YIG}}} - \alpha _{{\mathrm{YIG}}}} \right) = \frac{{4\pi M_{\mathrm{s}}{\mathrm{\Delta }}\tilde \alpha }}{{g\mu _{\mathrm{B}}}},$$

where *g* and *μ*_B_, are the Landé *g* factor and Bohr magneton, respectively. The maximum *g*_↑↓_ value (*d*_BS_ = 7 nm) is calculated to be ~2.2 × 10^15^ cm^−2^, about three times larger than that of a typical Pt/YIG sample. The large *g*_↑↓_ of Bi_2_Se_3_/YIG implies an efficient spin pumping to an excellent spin sink of Bi_2_Se_3_. Note that the trend in Fig. 2d is distinct from that of the normal metal (NM)/ferromagnetic metal (FM) structures. In NM/FM, the *g*_↑↓_ increases with increasing NM thickness as a result of vanishing spin backflow in thicker NM^34^. It is worth noting that the conducting bulk of Bi_2_Se_3_ can dissipate the spin-pumping-induced spin accumulation at the interface^6,35^. In this regard, the *d*_BS_ = 7 nm sample has the largest weight of surface state contribution to *g*_↑↓_. Such unconventional *d*_BS_ dependence of *g*_↑↓_ implies that TSS plays a dominant role in the damping enhancement.

### Spin-pumping signature of MPE and observation of the exchange effective field

**Fig. 3:**
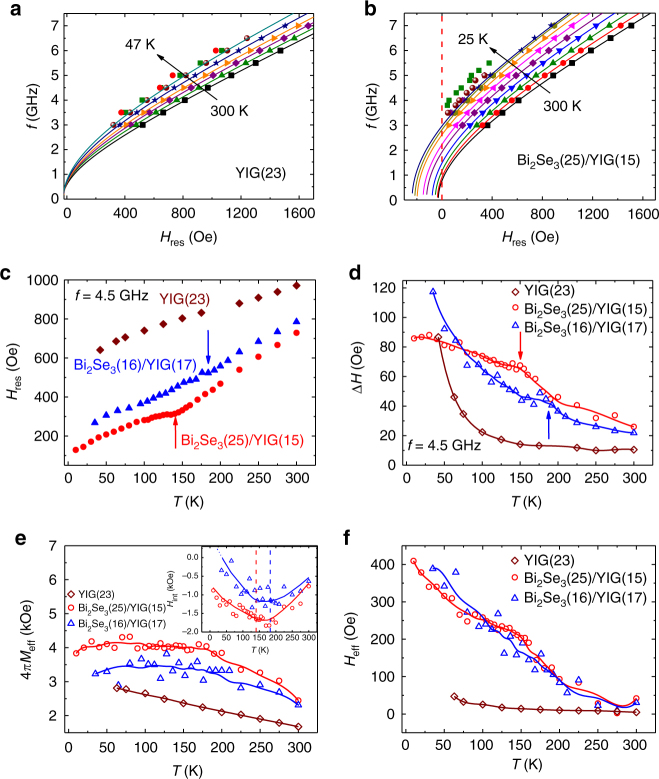
Temperature dependence of FMR characteristics of Bi_2_Se_3_/YIG. **a**, **b** *f* vs. *H*_res_ data for various *T* for YIG(23) and Bi_2_Se_3_(25)/YIG(15), respectively. Solids lines are fitted curves using Eq. (2). **c**, **d**, **e**, and **f** *T* dependence of *H*_res_, Δ*H*, 4*πM*_eff_, and *H*_eff_ of one YIG(23) single layer and two Bi_2_Se_3_/YIG bilayer samples, Bi_2_Se_3_(25)/YIG(15) and Bi_2_Se_3_(16)/YIG(17), respectively. The arrows in **c** and **d** denote the position of the hump-like features. Solid lines are guides of the eyes obtained by properly smoothing the experimental data. The inset of **e** shows the *T* dependence of *H*_int_. The dashed lines indicate the hump position shown in **c** and **d**

Since the effects of TSS are expected to enhance at low temperature, we next performed temperature-dependent FMR on Bi_2_Se_3_/YIG. Two bilayer samples Bi_2_Se_3_(25)/YIG(15) and Bi_2_Se_3_(16)/YIG(17), and a single layer YIG(23) were measured for comparison. Figure 3a, b shows the *H*_res_ vs. *f* data at various temperatures *T* for YIG(23) and Bi_2_Se_3_(25)/YIG(15). The *H*_res_ of both samples shows negative shifts at all *f* with decreasing *T*. The data of YIG(23) can be reproduced by the Kittel equation with increasing *M*_s_ of YIG at low *T*. In sharp contrast, Bi_2_Se_3_(25)/YIG(15) exhibited negative intercepts at *H*_res_, and the intercepts gained their magnitude when the sample was cooled down. This behavior of non-zero intercept is common for all of our Bi_2_Se_3_/YIG samples. Note that the Kittel equation in its original form cannot produce an intercept. To account for the behavior, a phenomenological effective field *H*_eff_ is added to the Kittel equation, i.e.,

2 $$f = \frac{\gamma }{{2\pi }}\sqrt {\left( {H_{{\mathrm{res}}} + H_{{\mathrm{eff}}}} \right)\left( {H_{{\mathrm{res}}} + H_{{\mathrm{eff}}} + 4\pi M_{{\mathrm{eff}}}} \right)}.$$

The solid lines in Fig. 3b generated by the modified Kittel equation fitted the experimental data very well.

Figure 3c, d presents the *T* dependence of *H*_res_ and Δ*H* for the YIG(23) and two Bi_2_Se_3_/YIG samples. As we lowered *T*, all of the samples had decreasing *H*_res_, which was viewed as the effect of the concurrently increasing *M*_eff_ and *H*_eff_ as seen in Fig. 3a, b. On the other hand, Δ*H* built up with decreasing *T*. We first examined Δ*H* of the YIG(23) single layer. The Δ*H* remained relatively unchanged with *T* decreasing from room temperature, and dramatically increased below 100 K. The pronounced *T* dependence of Δ*H* or *α* has been explored in various rare-earth iron garnet and was explained by the slow-relaxation process via rare-earth elements or Fe^2+^ impurities triggered at low *T*^36^. For sputtered YIG films, specifically, the increase in Δ*H* was less prominent in thicker YIG, indicating that the dominant impurities were located near the YIG surface^37^. Distinct from that of YIG(23), the Δ*H* progressively increased for the bilayer samples. We were not able to detect FMR signals with Δ*H* beyond 100 Oe due to the limited sensitivity of our co-planar waveguide. However, one can clearly see that, for Bi_2_Se_3_(25)/YIG(15) and Bi_2_Se_3_(16)/YIG(17), Δ*H* broadened owing to increased spin pumping at first. For Bi_2_Se_3_(25)/YIG(15), the Δ*H* curve gradually leveled off, and intersected with that of YIG(23) at *T* ~40 K. The seemingly antidamping by Bi_2_Se_3_ at low *T* may be related to the modification of the YIG surface chemistry during the Bi_2_Se_3_ deposition. Additional analyses are needed to verify the scenario, which is, however, beyond the scope of this work. For the Bi_2_Se_3_(10)/YIG and Bi_2_Se_3_(7)/YIG samples, the damping had increased to such large magnitude below 150 K, and FMR could not be easily detected.

In both *H*_res_ and Δ*H* curves, hump-like features located at *T* = 140 and 180 K (indicated by the arrows) were revealed for Bi_2_Se_3_(25)/YIG(15) and Bi_2_Se_3_(16)/YIG(17), respectively. We note that the humps are reminiscent of spin pumping into a fluctuating magnet close to its magnetic ordering temperature. As pointed out by Ohnuma et al.^38^, the spin-pumping efficiency is governed by the momentum sum of imaginary part of dynamical transverse spin susceptibility $\chi _k^R$ of the spin sink:

3 $$g_{ \uparrow \downarrow } \propto \mathop {\sum }\limits_k \frac{1}{{\omega _{{\mathrm{rf}}}}}{\mathrm{Im}}\chi _k^R\left( {\omega _{{\mathrm{rf}}}} \right),$$

where *k* is the wave vector and *ω*_rf_ is the microwave angular frequency. For a ferromagnet, the $\chi _k^R$ is known to be divergent near its *T*_C_^39^. Therefore, an enhancement of spin pumping is expected as the spin sink is close to its magnetic phase transition point^38,40,41^. In our system, a possibly newly formed magnetic phase would be the interfacial magnetization driven by the proximity effect, namely, *T*_C_ = 140 and 180 K for our Bi_2_Se_3_(25)/YIG(15) and Bi_2_Se_3_(16)/YIG(17), respectively. In fact, the *T*_C_ values of our samples are in good agreement with the reported *T*_C_ of 130 and 150 K in TI/YIG systems^14,22^.

Using Eq. (2), we further determine the *T* dependence of 4*πM*_eff_ and *H*_eff_ of YIG(23) and the two bilayers samples, as shown in Fig. 3e, f. The 4*πM*_eff_ of YIG(23) became larger monotonically as previously discussed, while the 4*πM*_eff_ of the bilayer samples increased before reaching a maximum when *T* was around 150 K, and then decreased slightly at low *T*. We further calculate the interfacial anisotropy field *H*_int_ using $4\pi M_{{\mathrm{eff}}}^{{\mathrm{BS}}/{\mathrm{YIG}}} - 4\pi M_{{\mathrm{eff}}}^{{\mathrm{YIG}}} \approx - H_{{\mathrm{int}}}$. The inset of Fig. 3e shows the *T* dependence of *H*_int_. The magnitude of *H*_int_ increased as the samples cooled down from room temperature at first. Upon crossing the temperature regions where the hump-like features are located, *H*_int_ magnitude started to decrease with further decreasing *T*. Although the samples exhibit interfacial IMA (*H*_int_ < 0) within the temperature range of our measurement, further extending the trend of Bi_2_Se_3_(16)/YIG(17), specifically, leads to interfacial PMA (*H*_int_  >0) below 40 K. The turning of *H*_int_ curves around 150 K implied that a competing magnetic anisotropy was emerging, which favored perpendicular direction and effectively diminished the IMA that persisted up to room temperature. Observing that the turning of *H*_int_ curves were in the vicinity of the individual hump temperature, we thus attribute the interfacial PMA to MPE in Bi_2_Se_3_/YIG. Our scenario is further supported by a theoretical model that considers the direct exchange coupling of TSS and an adjacent magnetic layer^31,42^. In this model, the calculated total electronic energy in the system with MPE indicates that PMA is in favor.

To independently show the effect of strong interfacial exchange coupling in Bi_2_Se_3_/YIG, we have performed electrical transport measurements at low *T*. As shown in Supplementary Fig. 7, we observed a clear negative magnetoresistance (MR) of Bi_2_Se_3_/YIG, which is distinct from weak antilocalization (WAL) effect typical of Bi_2_Se_3_ films without magnetic perturbation. Detailed analyses show that the MR data can be well reproduced if we assume that the TRS is broken and electrons are magnetically scattered at the bottom surface of Bi_2_Se_3_ (see Supplementary Note 4), which may be an indication of the presence of MPE in our Bi_2_Se_3_/YIG sample. However, we did not detect anomalous Hall effect in our samples, which might be obscured by the bulk conduction of Bi_2_Se_3_ in the transport measurements.

The *H*_eff_ of the bilayer samples, again, shows different *T* evolution than that of the bare YIG in Fig. 3f. *H*_eff_ built up with decreasing *T* in bilayers while the *H*_eff_ of the YIG single layer was *T* independent and close to zero. Phenomenologically, the *H*_eff_ resembles the exchange bias field of interlayer exchange coupling in an antiferromagnet/ferromagnet interface. However, we would like to exclude the possibility of exchange bias for the following two reasons. First, as shown in Supplementary Fig. 6, we did not observe shifts of magnetization hysteresis loop which is characteristic of an exchange bias effect^43^. Secondly, extending the field sweep to reversed applied field, we found that the FMR spectrum was symmetric with respect to the zero applied field, indicating that the direction of *H*_eff_ followed that of **M**. The observation is distinct from the magnetization pinning of exchange bias, in which the *H*_eff_ direction is fixed depending on the interfacial magnetic structure. The fact that *H*_eff_ existed only in FMR measurement suggests that it comes from spin-pumping-induced spin imbalance at the interface as previously reported^44^. Through exchange coupling to the magnetic layer, the non-equilibrium spin density 〈**S**〉_neq_ of the TSS gives rise to field-like torque:

4 $${\mathbf{T}}_{{\mathrm{FL}}} = {\mathrm{\Delta }}_{{\mathrm{ex}}}{\mathbf{M}} \times {\langle\mathbf{S}\rangle}_{{\mathrm{neq}}},$$

where Δ_ex_ is the exchange coupling constant^45^. ST-FMR experiments on NiFe/Bi_2_Se_3_^9^ and CoFeB/Bi_2_Se_3_^46^ showed large **T**_FL_ comparable to the damp-like torque owing to spin–momentum locking of TSS. Since spin pumping is the reciprocal process of ST-FMR, one can expect that the **T**_FL_ appears as an exchange effective field in spin pumping. Moreover, we noticed that the *T* dependence of *H*_eff_ in Fig. 3f resembles that of **T**_FL_ in CoFeB/Bi_2_Se_3_^46^, which implies that *H*_eff_ and **T**_FL_ share the same origin. Although a large **T**_FL_ can originate from other systems with strong SOC such as Rashba-split quantum well state^45^, which is likely to coexist with the TSS in Bi_2_Se_3_/YIG^47^, the **T**_FL_ from Rashba state is expected to decrease with decreasing Rashba coefficient at low *T*^48^. Here, we highlight that *H*_eff_ monotonically increased at low *T*. The unique *T* dependence of *H*_eff_ suggests that it is likely to originate from TSS.

### Zero-field FMR of Bi_2_Se_3_/YIG

**Fig. 4:**
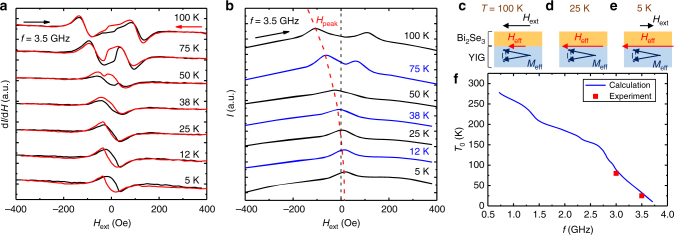
Zero-field FMR of the Bi_2_Se_3_(25)/YIG(15) sample. **a**, **b** FMR first derivative and microwave absorption spectra for various *T*, respectively. The arrows indicate the *H*_ext_ sweep direction. The dashed line in **b** traces the *T* evolution of the absorption peak *H*_peak_. **c**, **d**, and **e** Schematics of the Bi_2_Se_3_/YIG sample when *T* = 100, 25, and 5 K, respectively. **f** Zero-field FMR temperature *T*_0_ as a function of *f*

Finally, we demonstrated that the TSS-modulated magnetic anisotropy and *H*_eff_ in Bi_2_Se_3_/YIG are strong enough to induce FMR without an applied field *H*_ext_, which we term zero-field FMR. Figure 4a displays *T* evolution of FMR first derivative spectra of Bi_2_Se_3_(25)/YIG(15) at *f* = 3.5 GHz. The spectral shape started to deform when the *H*_res_was approaching zero. The sudden twists at *H*_ext_ ~ + 30 (−30) for positive (negative) field sweep arose from magnetization switching of YIG, and therefore led to hysteric spectra. The two spectra merged at 25 K and then separated again when *T* was further decreased. Figure 4b shows the microwave absorption intensity *I* spectra with positive field sweeps. We traced the peak position of *I* spectrum *H*_peak_ using the red dashed line, and found it coincided with zero *H*_ext_ at the zero-field FMR temperature *T*_0_ ~25 K. Below 25 K, *H*_peak_ moved across the origin and one needed to reverse *H*_ext_ to counter the internal effective field comprised of the demagnetization field 4*πM*_s_, *H*_int_, and *H*_eff_ (Fig. 4e). It should be pointed out that the presence of *H*_int_ alone would be inadequate to realize zero-field FMR. Only when *H*_eff_ is finite would the system exhibit intercepts as we have seen in Fig. 3b. We further calculate *T*_0_ as a function of microwave excitation frequency *f* (Fig. 4f) using Eq. (2) and the extracted *H*_eff_ of Fig. 3f. We obtain that, with finite *H*_eff_ persisting up to room temperature, zero-field FMR can be realized at high *T* provided *f* is sufficiently low. However, we emphasize that it is advantageous for YIG to be microwave-excited above 3 GHz. When *f* < 3 GHz, parasitic effects such as three-magnon splitting^49,50^ take place and significantly decrease the microwave absorption in YIG. Here, we demonstrate that the strong exchange coupling between Bi_2_Se_3_ and YIG gave rise to zero-field FMR in the feasible high frequency operation regime of YIG. Further improvement of interface quality of Bi_2_Se_3_/YIG is expected to raise *H*_eff_ and *T*_0_ for room-temperature, field free spintronic application.

## Discussion

Most experiments probing the spin transfer or spin–charge interconversion at TSS used FMs as the spin source/detector. The pitfall of FMs is that the constituent transition metals are chemically reactive with chalcogenides. Severe reactions can occur when an FM is deposited on a TI, forming new species that complicated the system under study. Even if an ideal TI/FM interface is achieved, theoretical study suggests that the electron doping from the FM can significantly shift the Fermi level of TIs and destroy the spin texture^27^. Besides, for SOT generation, current-shunting by FM reduces the current flowing in the TI and diminishes the SOT strength. Therefore, ferromagnetic insulators such as YIG is a far better platform to study the coupling mechanism between TSS and magnetic layers.

We attribute the high-temperature interfacial IMA to the enhanced exchange coupling of Fe^3+^ ions in YIG mediated by TSS based on the *d*_BS_ dependence of *K*_i_ in Fig. 2c. We emphasize that, although the model in ref. ^42^ predicts a PMA originated from direct exchange coupling between TSS and a magnetic layer, in reality, other contributions of magnetic anisotropy dependent on the detailed interfacial atomic structure can arise. As illustrated in ref. ^31^, in addition to the PMA from MPE, the stress anisotropy energy of EuS can also be magnified by the strong SOC of Bi_2_Se_3_, which would not necessarily be PMA for a material system other than EuS/Bi_2_Se_3_. Other factors such as the Fermi energy of Bi_2_Se_3_ can have pronounced effects on the exchange coupling constant and total anisotropy energy^31^. Given the multiple sources of magnetic anisotropy that are possibly influenced by TSS, an in-depth theoretical study will be needed to precisely describe the high-temperature interfacial IMA and the emerging low-temperature PMA of Bi_2_Se_3_/YIG.

The TSS-modulated magnetization dynamics presented in this work have important implications. Firstly, the electronic structure of TI/ferromagnetic insulator interface has a pronounced influence on the magnetization dynamics. It should be noted that the strong coupling between the TSS and YIG can potentially modify the TSS of pure Bi_2_Se_3_. Since the spin texture of TSS is critical for spin transport, an insertion layer may be needed to decouple YIG and Bi_2_Se_3_ for spintronics devices. The interface structure, in turn, depends strongly on the sample fabrication process. For example, Wang et al.^8^ reported a markedly different *d*_BS_ dependence of *g*_↑↓_ from the one shown in Fig. 2d. Specifically, our samples show larger $\Delta \tilde \alpha$ when *d*_BS_ was approaching the 2D limit. Note that the linewidth broadening observed in this work is overall larger than that reported in ref. ^8^ mainly because we have chosen thinner YIG films. The discrepancy in the *d*_BS_ dependence of *g*_↑↓_ may be reconciled by the different sample characteristics by comparing the TEM images and the surface morphology of Bi_2_Se_3_, etc.

Secondly, although the interface spin structure is of great interest to investigate, it has been difficult to measure with spin-polarized photoemission techniques because of the limited probing depth. Spin pumping provides another route to resolve the problem, since it has proven to be a powerful tool to probe magnetic phase transition of ultrathin films^40,41^. Here, we extended the concept and used spin pumping to study the MPE in Bi_2_Se_3_/YIG. The indicators of MPE are shown in Fig. 3c, d. Further testing of the validity of this method will depend on the improvements in the sample quality, such as a sharper interface and lowering the carrier density of Bi_2_Se_3_.

Lastly, the observations of large *K*_i_, $\Delta \tilde \alpha$ at room temperature, and *H*_eff_ at low temperature in Bi_2_Se_3_/YIG echo the theoretical predictions of the magnetization dynamics of a perpendicularly magnetized layer interacting with TSS^51–53^. According to these models, the gap opening of TSS due to broken TRS leads to topological (inverse) spin galvanic effect^51,52^, anisotropic shifts of FMR frequency^52^, and anisotropic damping^53^. Despite the fact that an interfacial PMA showed up at low temperature in Bi_2_Se_3_/YIG, the bilayer sample still exhibited a gross in-plane anisotropy due to the shape anisotropy of YIG. However, the notable modulation of the YIG properties presented in this work is a promising start to examine these models. We expect ferromagnetic insulators with PMA, such as strained TmIG^54^, will offer new opportunities to realize the phenomena.

In summary, we have investigated the magnetization dynamics of YIG in the presence of interfacial exchange coupling and TSS of Bi_2_Se_3._ The significantly modulated magnetization dynamics at room temperature are shown to be TSS-originated through the Bi_2_Se_3_ thickness dependence study. The temperature-dependent study reveals a possible signature of MPE and an emerging PMA that compensates the high-temperature IMA, with a spin-pumping-induced effective field increasing toward low temperature. The underlying mechanism of these phenomena calls for further theoretical modeling and understanding. To our knowledge, this is the first work that links the magnetization dynamics of the magnetic layer to TSS, showing that FMR and spin pumping can be effective techniques to probe the interface magnetic properties. Moreover, the TSS-modulated dynamics are a cornerstone for future investigation on novel physics such as topological inverse spin galvanic effect, and further raise several interesting topics. For example, how the *H*_eff_, a quantity that comes from the non-equilibrium process of spin pumping, depends on the spin texture of TSS and the interfacial magnetic anisotropy will be an important question to answer. Temperature-dependent FMR with out-of-plane setup should provide us with valuable information. Therefore, understanding the interplay between these phenomena and further manipulating them will be a step forward toward developing TI-based spintronics.

## Methods

### Sample preparation and structural properties

The YIG thin films were deposited on (111)-oriented gadolinium gallium garnet (GGG) substrates by off-axis sputtering at room temperature. The GGG(111) substrates were first ultrasonically cleaned in order of acetone, ethanol, and DI-water before being mounted in a sputtering chamber with the base pressure of 2 × 10^−7^ Torr. For YIG deposition, a 2-inch YIG target was sputtered with the following conditions: an applied rf power of 75 W, an Ar pressure of 50 mtorr, and a growth rate of 0.6 nm/min. The samples were then annealed at 800 °C with an O_2_ pressure of 11.5 mtorr for 3 h. Supplementary Fig. 1(a) displays the atomic force microscopy (AFM) image of the YIG surface, showing a flat surface with a roughness of 0.19 nm. Supplementary Fig. 1(b) shows the high-angle annular dark-field (HAADF) image of YIG/GGG. The YIG thin film was epitaxially grown on the GGG substrate with excellent crystallinity. No crystal defects were observed at the YIG bulk and YIG/GGG interface.

The YIG/GGG samples were annealed at 450 °C in the MBE growth chamber for 30 min prior to Bi_2_Se_3_ growth at 280 °C. The base pressure of the system was kept about 2 × 10^−10^ Torr. Elemental Bi (7N) and Se (7N) were evaporated from regular effusion cells^55^. As shown in Supplementary Fig. 3(a), streaky reflection high-energy electron diffraction (RHEED) patterns of Bi_2_Se_3_ were observed. Supplementary Fig. 3(b) displays the surface morphology of 7 quintuple layer (QL) Bi_2_Se_3_ taken by AFM. The image shows layer-by-layer growth of Bi_2_Se_3_ with the step heights ~1 nm, which corresponds to the thickness of 1 QL. The surface roughness of our 7 QL Bi_2_Se_3_ is ~0.28 nm within a layer. The layer structure of Bi_2_Se_3_ was also revealed by the HAADF image shown in Supplementary Fig. 3(c). Despite the high-quality growth of Bi_2_Se_3_, an amorphous interfacial layer of ~1 nm formed. The excellent crystallinity of our samples was verified by clear Pendellösung fringes of the synchrotron radiation x-ray diffraction (SR-XRD) data shown in Supplementary Fig. 3(d). The fringes of YIG(444) peak do not show clear changes before and after the growth of Bi_2_Se_3_, indicating that the lattice parameter in the normal direction of YIG remains unchanged. To check the lattice parameter of in-plane direction, we also performed in-plane radial scans of YIG/GGG(22–4) peaks. Supplementary Fig. 3(e) shows that the peaks position measured before and after growing Bi_2_Se_3_ is perfectly matched, indicating the absence of Bi_2_Se_3_-induced strains in YIG that might contribute additional magnetic anisotropy^56^. Supplementary Fig. 3(f) displays the x-ray reflectivity (XRR) data of our Bi_2_Se_3_(6)/YIG(50) sample. From the fit to the data, we extract the Bi_2_Se_3_ surface roughness and Bi_2_Se_3_/YIG interface roughness to be 0.16 nm and 0.22 nm, respectively. Note that the interface roughness of Bi_2_Se_3_/YIG is close to that of YIG surface (0.19 nm), which means the interdiffusion at the interface is at minimal, if any.

### FMR measurement setup

To investigate the magnetic properties of Bi_2_Se_3_/YIG, room-temperature angle- and frequency-dependent FMR measurements were performed independently using a cavity and co-planar waveguide, respectively (Fig. 1a, b). For the temperature-dependent FMR, the co-planar waveguide was mounted in a cryogenic probe station (Lake Shore, CPX-HF), which enables samples to be cooled as low as 5 K. The external field is modulated for lock-in detection in all of the measurements. The modulation amplitude was kept below 1/4 of the FMR linewidth to avoid serious spectral distortions. The microwave source power was no larger than 5 dBm.

### Data availability

The experimental data of this work are available from the corresponding authors upon reasonable request.

## Electronic supplementary material

Supplementary Information

Peer Review File

## References

[1] Sinova J (2015). Spin Hall effects. Rev. Mod. Phys..

[2] Hasan MZ, Kane CL (2010). Colloquium: topological insulators. Rev. Mod. Phys..

[3] Qi XL, Zhang SC (2011). Topological insulators and superconductors. Rev. Mod. Phys..

[4] Hsieh D (2009). A tunable topological insulator in the spin helical Dirac transport regime. Nature.

[5] Xu S (2011). Topological phase transition and texture inversion in a tunable topological insulator. Science.

[6] Shiomi Y (2014). Spin-electricity conversion induced by spin injection into topological insulators. Phys. Rev. Lett..

[7] Rojas-Sánchez JC (2016). Spin to charge conversion at room temperature by spin pumping into a new type of topological insulator: α-Sn films. Phys. Rev. Lett..

[8] Wang H (2016). Surface-state-dominated spin-charge current conversion in topological-insulator-ferromagnetic-insulator heterostructures. Phys. Rev. Lett..

[9] Mellnik AR (2014). Spin-transfer torque generated by a topological insulator. Nature.

[10] Jamali M (2015). Giant spin pumping and inverse spin hall effect in the presence of surface and bulk spin-orbit coupling of topological insulator Bi_2_Se_3_. Nano Lett..

[11] Jiang Z (2016). Enhanced spin Seebeck effect signal due to spin-momentum locked topological surface states. Nat. Commun..

[12] Han J (2017). Room-temperature spin-orbit torque switching induced by a topological insulator. Phys. Rev. Lett..

[13] Wei P (2013). Exchange-coupling-induced symmetry breaking in topological insulators. Phys. Rev. Lett..

[14] Lang M (2014). Proximity induced high-temperature magnetic order in topological insulator—ferrimagnetic insulator heterostructure. Nano Lett..

[15] Katmis F (2016). A high-temperature ferromagnetic topological insulating phase by proximity coupling. Nature.

[16] Chen YL (2010). Massive Dirac fermion on the surface of a magnetically doped topological insulator. Science.

[17] Qi XL, Hughes TL, Zhang SC (2008). Topological field theory of time-reversal invariant insulators. Phys. Rev. B.

[18] Chang CZ (2013). Experimental observation of the quantum anomalous hall effect in a magnetic topological insulator. Science.

[19] Yu R (2010). Quantized anomalous hall effect in magnetic topological insulators. Science.

[20] Li M (2015). Proximity-driven enhanced magnetic order at ferromagnetic-insulator-magnetic-topological-insulator interface. Phys. Rev. Lett..

[21] Lee C, Katmis F, Jarillo-Herrero P, Moodera JS, Gedik N (2016). Direct measurement of proximity-induced magnetism at the interface between a topological insulator and a ferromagnet. Nat. Commun..

[22] Jiang Z (2015). Independent tuning of electronic properties and induced ferromagnetism in topological insulators with heterostructure approach. Nano Lett..

[23] Sun Y (2013). Damping in yttrium iron garnet nanoscale films capped by platinum. Phys. Rev. Lett..

[24] Wang HL (2014). Scaling of spin hall angle in 3d, 4d, and 5d metals from Y_3_Fe_5_O_12_/metal spin pumping. Phys. Rev. Lett..

[25] Tserkovnyak Y, Brataas A, Bauer GEW (2002). Enhanced Gilbert damping in thin ferromagnetic films. Phys. Rev. Lett..

[26] Zhang Y (2010). Crossover of the three-dimensional topological insulator Bi_2_Se_3_ to the two-dimensional limit. Nat. Phys..

[27] Zhang J, Velev JP, Dang X, Tsymbal EY (2016). Band structure and spin texture of Bi_2_Se_3_ 3d ferromagnetic metal interface. Phys. Rev. B.

[28] Men’shov VN, Tugushev VV, Eremeev SV, Echenique PM, Chulkov EV (2013). Magnetic proximity effect in the three-dimensional topological insulator/ferromagnetic insulator heterostructure. Phys. Rev. B.

[29] Xia Y (2009). Observation of a large-gap topological-insulator class with a single Dirac cone on the surface. Nat. Phys..

[30] Kajiwara Y (2010). Transmission of electrical signals by spin-wave interconversion in a magnetic insulator. Nature.

[31] Kim J, Kim KW, Wang H, Sinova J, Wu R (2017). Understanding the giant enhancement of exchange interaction in Bi_2_Se_3_-EuS heterostructures. Phys. Rev. Lett..

[32] Neupane M (2014). Observation of quantum-tunnelling-modulated spin texture in ultrathin topological insulator Bi_2_Se_3_ films. Nat. Commun..

[33] Landolt G (2014). Spin texture of Bi_2_Se_3_ thin films in the quantum tunneling limit. Phys. Rev. Lett..

[34] Tserkovnyak Y, Brataas A, Bauer GEW, Halperin BI (2005). Nonlocal magnetization dynamics in ferromagnetic heterostructures. Rev. Mod. Phys..

[35] Yamamoto KT, Shiomi Y, Segawa K, Ando Y, Saitoh E (2016). Universal scaling for the spin-electricity conversion on surface states of topological insulators. Phys. Rev. B.

[36] Seiden PE (1964). Ferrimagnetic resonance relaxation in rare-earth iron garnets. Phys. Rev..

[37] Jermain CL (2017). Increased low-temperature damping in yttrium iron garnet thin films. Phys. Rev. B.

[38] Ohnuma Y, Adachi H, Saitoh E, Maekawa S (2014). Enhanced dc spin pumping into a fluctuating ferromagnet near T_C_. Phys. Rev. B.

[39] Hohenberg PC, Halperin BI (1977). Theory of dynamic critical phenomena. Rev. Mod. Phys..

[40] Frangou L (2016). Enhanced spin pumping efficiency in antiferromagnetic IrMn thin films around the magnetic phase transition. Phys. Rev. Lett..

[41] Qiu Z (2016). Spin-current probe for phase transition in an insulator. Nat. Commun..

[42] Semenov YG, Duan X, Kim KW (2012). Electrically controlled magnetization in ferromagnet-topological insulator heterostructures. Phys. Rev. B.

[43] Manna PK, Yusuf SM (2014). Two interface effects: exchange bias and magnetic proximity. Phys. Rep..

[44] Abdulahad FB (2015). Spin chemical potential bias induced surface current evidenced by spin pumping into the topological insulator Bi_2_Te_3_. Phys. Rev. B.

[45] Fischer MH, Vaezi A, Manchon A, Kim EA (2016). Spin-torque generation in topological insulator based heterostructures. Phys. Rev. B.

[46] Wang Y (2015). Topological surface states originated spin-orbit torques in Bi_2_Se_3_. Phys. Rev. Lett..

[47] Bahramy MS (2012). Emergent quantum confinement at topological insulator surfaces. Nat. Commun..

[48] Eldridge PS (2008). All-optical measurement of Rashba coefficient in quantum wells. Phys. Rev. B.

[49] Suhl S (1957). The theory of ferromagnetic resonance at high signal power. J. Phys. Chem. Solids.

[50] Iguchi R (2012). Spin pumping without three-magnon splitting in polycrystalline Bi_1_Y_2_Fe_5_O_12_/Pt bilayer structure. Jpn. J. Appl. Phys..

[51] Garate I, Franz M (2010). Inverse spin-galvanic effect in the interface between a topological insulator and a ferromagnet. Phys. Rev. Lett..

[52] Yokoyama T, Zang J, Nagaosa N (2010). Theoretical study of the dynamics of magnetization on the topological surface. Phys. Rev. B.

[53] Ndiaye PB (2017). Dirac spin-orbit torques and charge pumping at the surface of topological insulators. Phys. Rev. B.

[54] Tang C (2016). Anomalous Hall hysteresis in Tm_3_Fe_5_O_12_/Pt with strain-induced perpendicular magnetic anisotropy. Phys. Rev. B.

[55] Chen KHM (2017). Van der Waals epitaxy of topological insulator Bi_2_Se_3_ on single layer transition metal dichalcogenide MoS_2_. Appl. Phys. Lett..

[56] Wang HL, Du CH, Hammel PC, Yang F (2014). Strain-tunable magnetocrystalline anisotropy in epitaxial Y_3_Fe_5_O_12_ thin films. Phys. Rev. B.

