# Supplementary material for: Strongly exchange-coupled and surface-state-modulated magnetization dynamics in Bi2Se3/yttrium iron garnet heterostructures
Source: Nat Commun. 2018 Jan 15;9:223. doi: 10.1038/s41467-017-02743-2 (PMC5768741; doi:10.1038/s41467-017-02743-2)
Supplement: Supplementary file 1 — Supplementary Information [file 41467_2017_2743_MOESM1_ESM.pdf]

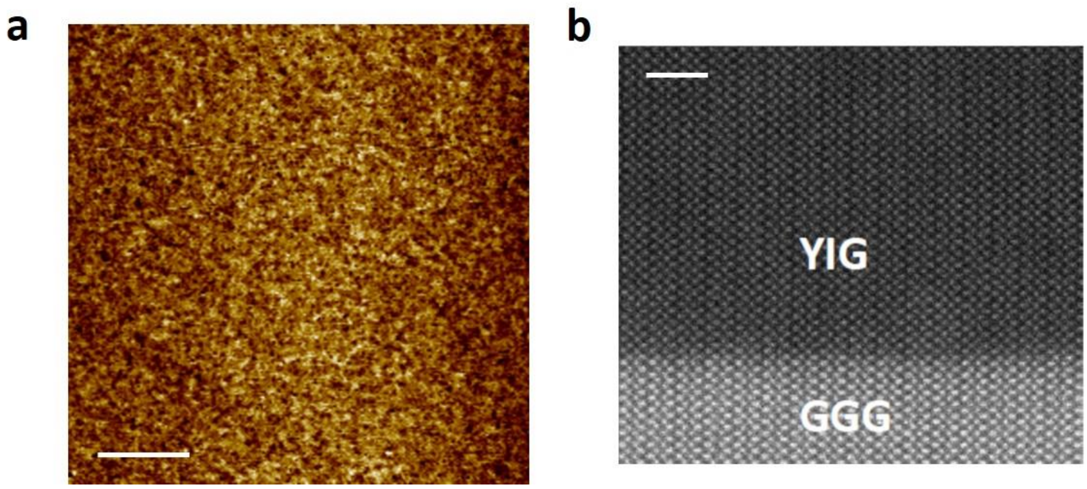

Supplementary Figure 1 | Surface morphology and the HAADF-STEM image of a representative sputtered YIG film grown on GGG(111) substrates. (a) AFM surface image, with the scale bar representing 1 μm. The surface roughness is ~ 0.19 nm. (b) HAADF-STEM image of YIG/GGG. The scale bar represents 2 nm.

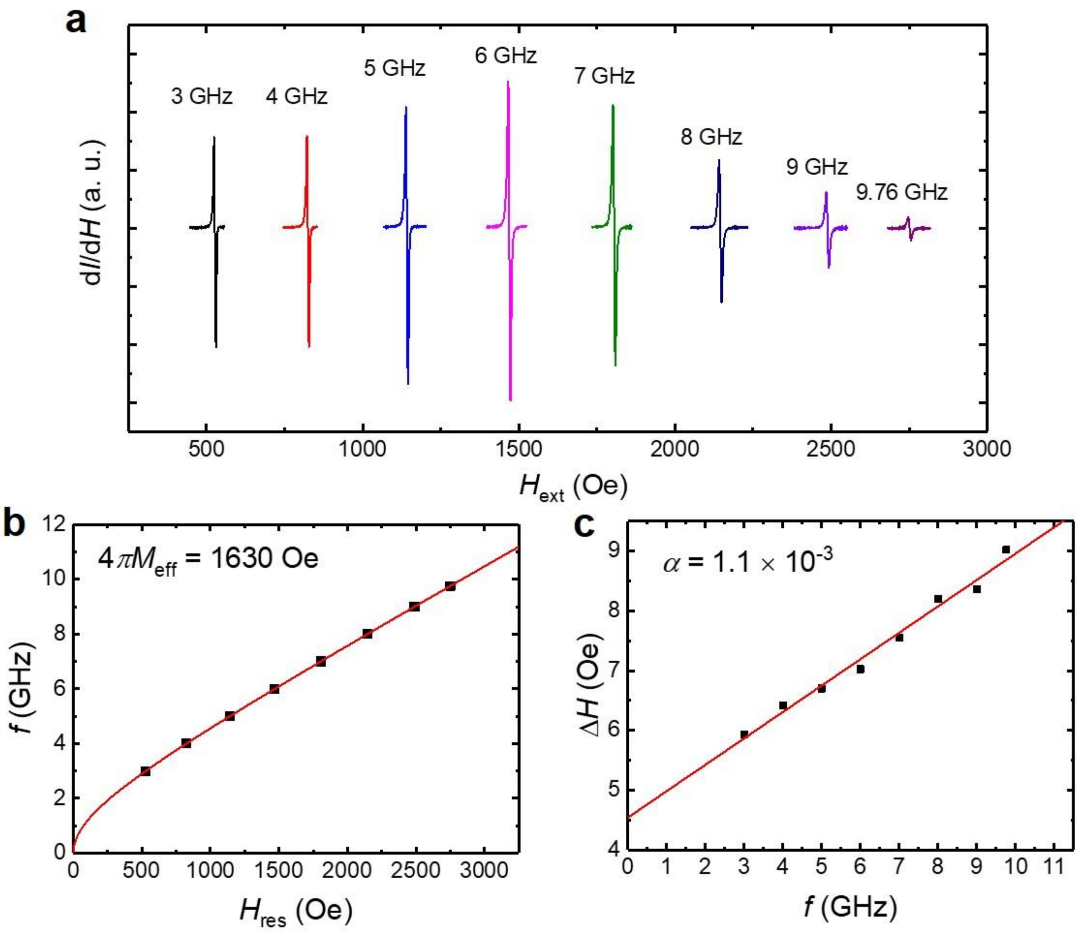

Supplementary Figure 2 | Representative FMR properties of YIG thin films obtained with a coplanar wave guide. (a) FMR first-derivative spectra of 23 nm YIG at various frequencies. (b) f vs H_{res} data fitted to the Kittel equation (red line). (c) ΔH as a function of f data. From the linear fit (red line) the α value of the sample is obtained.

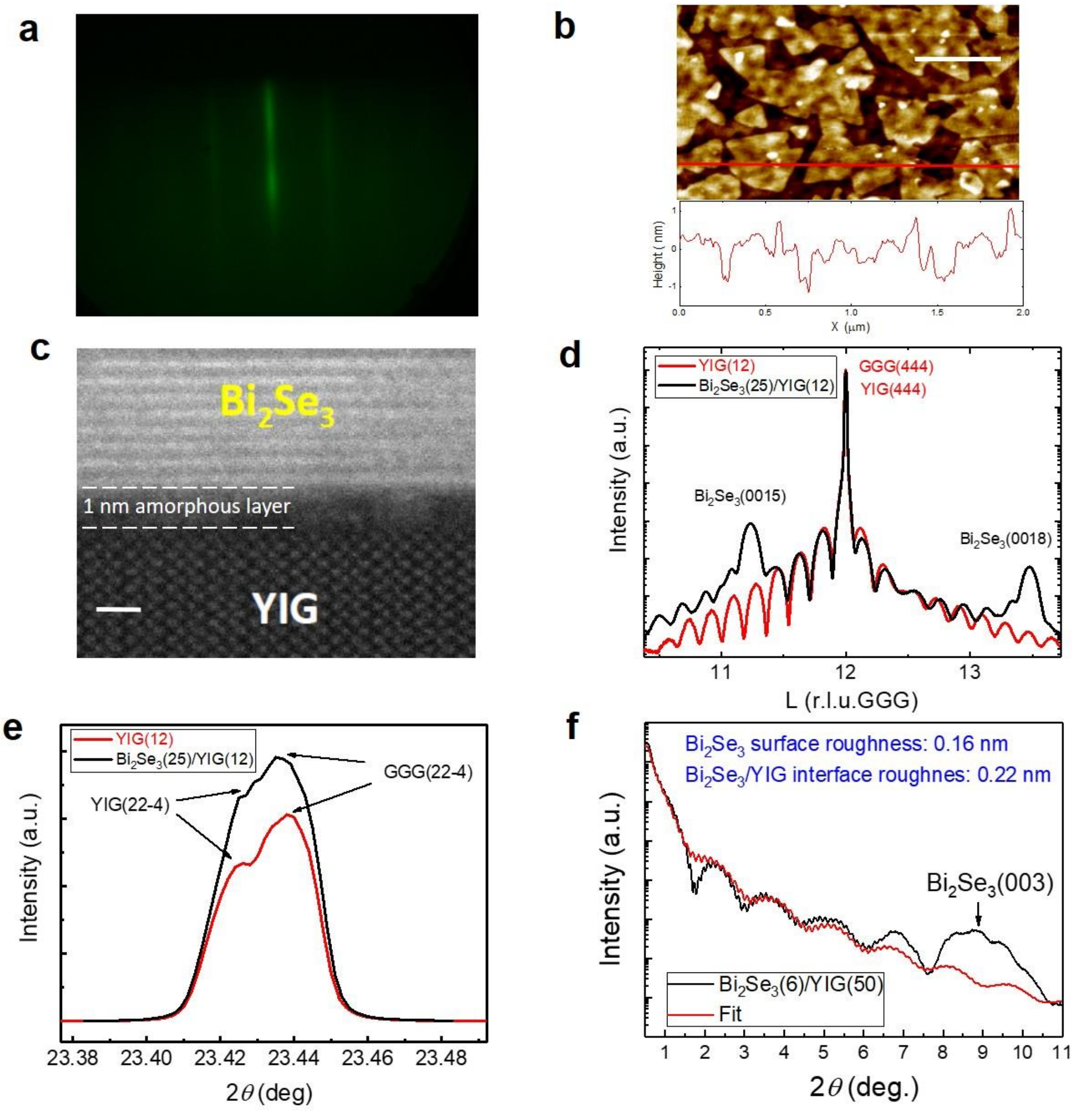

Supplementary Figure 3 | Structural characterizations of Bi_2Se_3/YIG samples. (a) RHEED patterns of MBE grown 7 QL Bi_2Se_3 on YIG/GGG(111) substrates. (b) AFM image of a 7 QL Bi_2Se_3. The scale bar is 500 nm. (c) HAADF-STEM image of Bi_2Se_3/YIG/GGG heterostructures. The scale bar represents 1 nm.(d) SR-XRD of our Bi_2Se_3(25)/YIG(12) sample. Clear Pendellösung fringes of YIG and Bi_2Se_3 indicates excellent crystallinity. (e) In-plane radial scan of YIG(12) and Bi_2Se_3(25)/YIG(12). The peak at 23.44° is attributed to GGG(22-4), whereas the shoulder to YIG(22-4). (f) XRR results of Bi_2Se_3(6)/YIG(50) and the

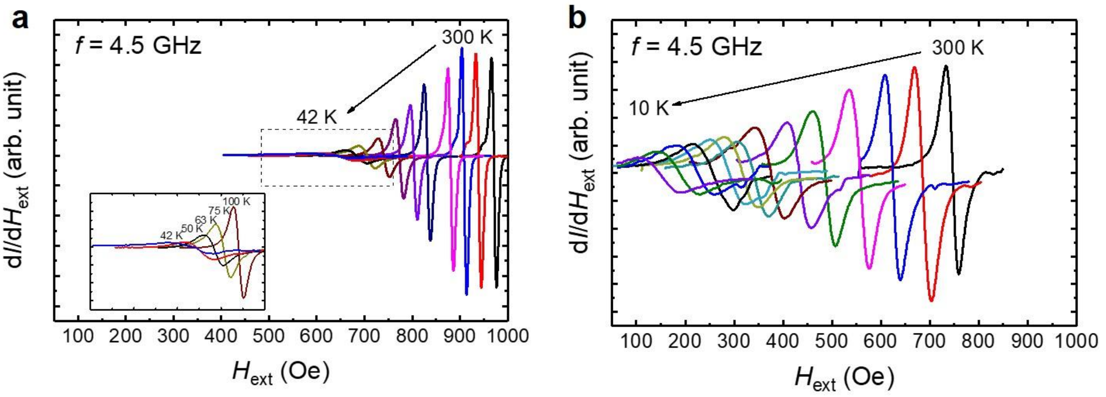

Supplementary Figure 4 | Temperature-dependent FMR first-derivative spectra of the YIG single layer and Bi_2Se_3/YIG bilayer sample. (a) FMR spectra at various temperatures of YIG(23). (b) FMR spectra at various temperatures of Bi_2Se_3(25)/YIG(15). The ΔH increased with decreasing T, accompanied by decreased dI/dH_{ext} peak magnitudes because of enhanced damping. The H_{ext} scale are fixed to clearly show the pronounced changes of H_{res} and ΔH induced by Bi_2Se_3.

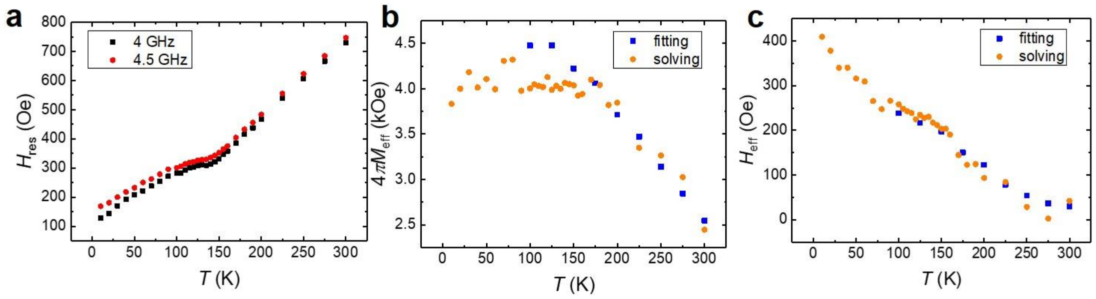

Supplementary Figure 5 | Illustration of extraction of $4\pi M_{\mathrm{eff}}$ and H_{eff} from temperature dependence H_{res} data. (a) H_{res} vs T data of Bi_2Se_3(25)/YIG(15) measured at 4 and 4.5 GHz. (b) and (c) Comparison of extracted $4\pi M_{\mathrm{eff}}$ and H_{eff} by the fitting and solving method.

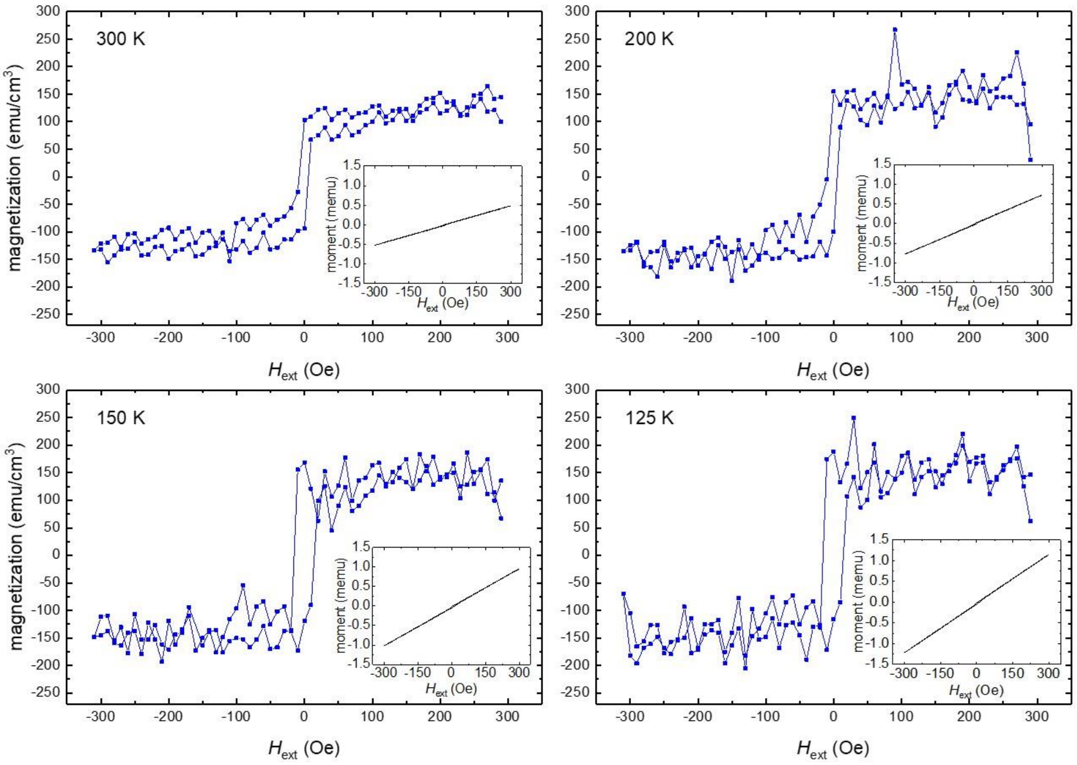

Supplementary Figure 6 | Temperature dependence of magnetization hysteresis loop of Bi_2Se_3(16)/YIG(17) measured by a SQUID magnetometer. The paramagnetic background of the GGG substrate has been subtracted. No shifts of hysteresis loops were observed. Insets are raw data showing large paramagnetic GGG signals that increases with decreasing *T*.

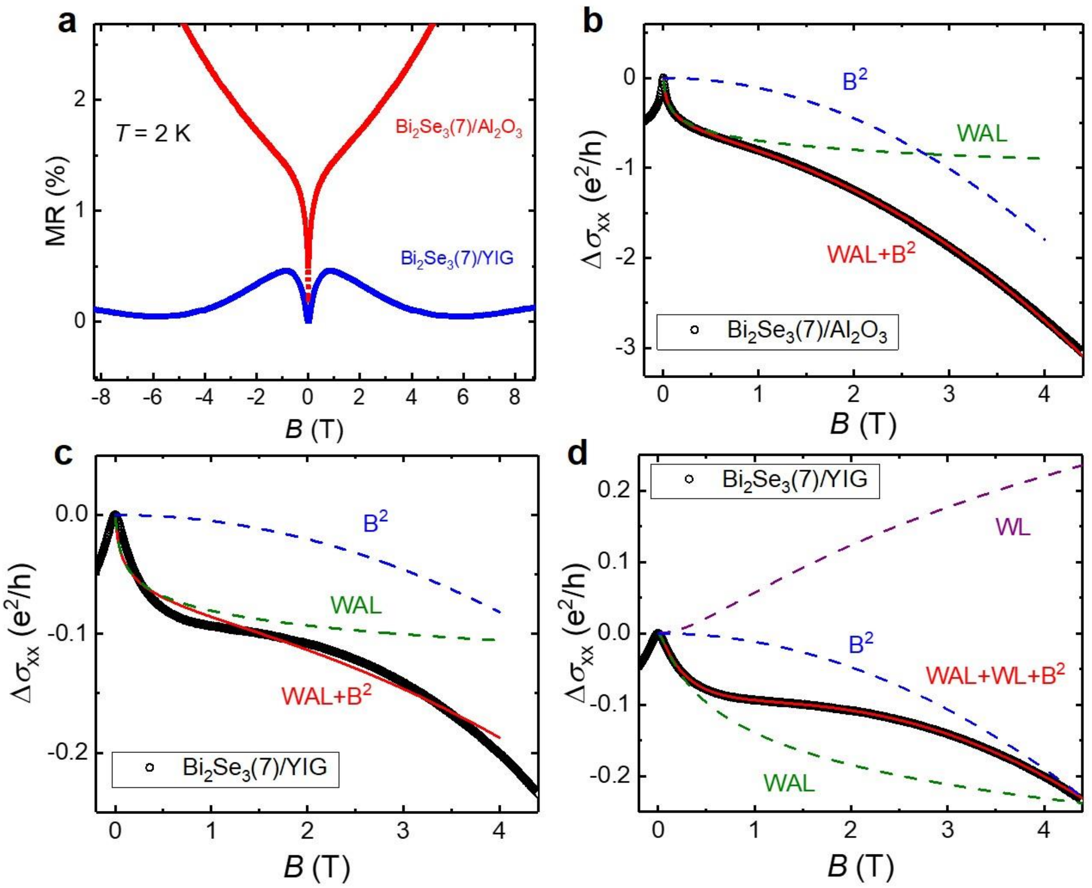

Supplementary Figure 7 | Magnetoresistance of Bi_2Se_3/YIG and data analyses. (a) Magnetoresistance of Bi_2Se_3(7)/YIG and Bi_2Se_3(7)/Al_2O_3. MR is defined as $\left(\rho_{xx}(B) - \rho_{xx}(0)\right)/\rho_{xx}(0)$. (b) Magnetoconductance (MC) of Bi_2Se_3(7)/Al_2O_3 fitted to Eq. S5 containing WAL and B^2 (WAL+ B^2) components. (c) and (d) MC of Bi_2Se_3(7)/YIG fitted to WAL+B^2 and WAL+WL+B^2.

Supplementary Table 1 | Fittng results of Supplementary Figure 7(b) and (d) using Supplementary Eq. 5.

	α_0	$l_{\phi 0}$ (nm)	α_1	$l_{\phi 1}$ (nm)	c (10^{-7})
Bi_2Se_3(7)/Al_2O_3	N/A	N/A	-0.456 ± 0.001	380 ± 2	-43.53 ± 0.003
Bi_2Se_3(7)/YIG	0.690 ± 0.002	24.2 ± 0.1	-0.221 ± 0.003	87.4 ± 0.3	-4.592 ± 0.009

Supplementary Note 1: FMR characteristics of the sputtered YIG films

Supplementary Fig. 2(a) shows the representative FMR data of our YIG film measured by a coplanar waveguide. The FMR spectra exhibit Lorentzian lineshape at all measured frequencies ranging from 3 to 9.76 GHz. To determine the $4\pi M_{\mathrm{eff}}$ and α, the resonance fields H_{res} and peak-to-peak widths ΔH of these spectra were plotted as a function of f as shown in Supplementary Fig. 2(b) and (c), respectively. We obtain the $4\pi M_{\mathrm{eff}}$ of our 23 nm YIG to be 1630 Oe. We note that the $4\pi M_{\mathrm{eff}}$ value is lower than the reported values of the YIG prepared by either sputtering or pulsed laser deposition[1-3]. The difference may come from growth conditions dependent on different systems. For determination of α, the data in Supplementary Fig. 2(c) is fitted to the following equation,

$$\Delta H = \Delta H_0 + \frac{4\pi f \alpha}{\sqrt{3}\gamma}. \quad (1)$$

Here, ΔH_0 and γ are the inhomogeneous broadening and gyromagnetic ratio. The linear fit in Supplementary Fig. 2(c) corresponds to $\alpha = 1.1 \times 10^{-3}$.

Supplementary Note 2: Analyses of magnetic anisotropy

We express the free energy density E of the system as

$$E = -\mathbf{M} \cdot \mathbf{H} + \frac{1}{2} M_{\mathrm{s}} (4\pi M_{\mathrm{s}} - H_{\mathrm{an}} - H_{\mathrm{int}}) \cos^2\theta_{\mathrm{M}} \quad (2)$$

, where $\mathbf{M}$, $\mathbf{H}$, M_{s}, H_{an} and θ_{M} are magnetization vector, applied field vector, saturation magnetization, the anisotropy field induced by Bi_2Se_3 and magnetization angle with respect to the surface normal, respectively. We further define the effective demagnetization field $4\pi M_{\mathrm{eff}} = 4\pi M_{\mathrm{s}} - H_{\mathrm{an}} - H_{\mathrm{int}}$, where H_{an} is the magnetocrystalline anisotropy field of sputtered YIG and H_{int} is the interfacial anisotropy field. We have $H_{\mathrm{int}} = 0$ for the YIG single layer by definition. The first term of Supplementary Eq. (2) is the Zeeman energy and the second term accounts for

uniaxial out-of-plane anisotropy. Here, we neglect higher order terms that are relatively small for a strain-free cubic system. The H_{res} can be calculated by minimizing E and, at the equilibrium angle of **M,** solving the Smit-Beljers equation[4],

$$\left(\frac{\omega}{\gamma}\right)^2 = \frac{1}{M^2 \sin^2 \theta_{\mathrm{M}}}\left[\frac{\partial^2 E}{\partial \theta_{\mathrm{M}}^2}\frac{\partial^2 E}{\partial \varphi_{\mathrm{M}}^2} - \left(\frac{\partial^2 E}{\partial \theta_{\mathrm{M}}^2\, \partial \varphi_{\mathrm{M}}^2}\right)^2\right] \tag{3}$$

As $\theta_{\mathrm{H}} = \pi/2$, the FMR conditions reduce to the Kittel equation $f = \frac{\gamma}{2\pi}\sqrt{H_{\mathrm{res}}(H_{\mathrm{res}} + 4\pi M_{\mathrm{eff}})}$.

We can safely assume that the H_{an} did not change before and after the growth of Bi_2Se_3 based on Supplementary Fig. 3(d) and (e). With this in mind, we further notice that K_{i} can be alternatively expressed as $(1/2)\left(4\pi M_{\mathrm{eff}}^{\mathrm{YIG}} - 4\pi M_{\mathrm{eff}}^{\mathrm{BS/YIG}}\right) M_{\mathrm{s}} d_{\mathrm{YIG}}$, where $4\pi M_{\mathrm{eff}}^{\mathrm{YIG}}$ $\left(4\pi M_{\mathrm{eff}}^{\mathrm{BS/YIG}}\right)$ represents the $4\pi M_{\mathrm{eff}}$ of YIG (Bi_2Se_3/YIG) for a specific d_{YIG}. The calculated K_{i} using this expression for d_{BS} = 25 nm gives an average of -0.068 erg/cm^{-2}, in good agreement with a K_{i} of -0.075 erg/cm^2 obtained from the linear fit in Fig. 2(b).

Supplementary Note 3: Extraction of $4\pi M_{\mathrm{eff}}$ and H_{eff} from temperature dependence of H_{res}

Since the effective damping constant of YIG and Bi_2Se_3/YIG increased pronouncedly at low T, the weakened FMR signal was inevitably accompanied by larger uncertainties of measured H_{res}. The uncertainties are even more serious when $T < 150$ K and $f > 5$ GHz for our measurements. Fitting the data including points in the $f > 5$ GHz region to the Kittel equation gives large errors of $4\pi M_{\mathrm{eff}}$ and H_{eff}, which obscure the temperature dependency of these two quantities. To reduce the uncertainties in the process of extracting $4\pi M_{\mathrm{eff}}$ and H_{eff}, we focused on the FMR data for $f = 4$ and 4.5 GHz, from which we obtained H_{res} with satisfactory accuracy (Supplementary Fig. 5(a)). The Kittel equation can be arranged in the

following form,

$$H_{\rm eff}^2 + (2H_{\rm res} + 4\pi M_{\rm eff})H_{\rm eff} + \left(4\pi M_{\rm eff}H_{\rm res} + H_{\rm res}^2 - \frac{4\pi^2 f^2}{\gamma^2}\right) = 0. \qquad (4)$$

With $\gamma = 1.77 \times 10^{11}\ \mathrm{t^{-1}s^{-1}}$, the two sets of data in Supplementary Fig. 5(a) provided us with sufficient information to explicitly solve the second-order equation. Supplementary Fig. 5(b) and (c) compare the results of fitting the data and solving the Kittel equation. For $T > 150$ K, where FMR can be accurately measured up to 7 GHz, the $4\pi M_{\rm eff}$ and $H_{\rm eff}$ obtained from solving and fitting method agree well, demonstrating the reliability of the solving method.

Supplementary Note 4: Analyses of magnetoresistance data of Bi_2Se_3/YIG and Bi_2Se_3/Al_2O_3

Supplementary Fig. 7(a) displays the MR of Bi_2Se_3(7)/YIG and $Bi_2Se_3(7)/Al_2O_3$. A cusp-shape positive MR was observed for $Bi_2Se_3(7)/Al_2O_3$, which is the feature of WAL effect. In sharp contrast, a negative MR was detected at the field interval of 1 T to 5 T for Bi_2Se_3(7)/YIG. Since the major difference between the two samples is the magnetic properties of substrates (YIG is ferrimagnetic while Al_2O_3 is non-magnetic) that contact with the bottom surface of Bi_2Se_3, it is clear that the interfacial interaction of Bi_2Se_3/YIG play an important role in the peculiar MR.

The pronounced change of low-field MR or magnetoconductance (MC) of Bi_2Se_3 thin films has been commonly attributed to WAL effect. In quantum diffusive transport regime (electron mean free path $l \ll$ phase coherence length l_Φ), the surface state electrons travelling a time-reversed loops around the Fermi surface gain π Berry phase. The destructive quantum interference then prohibits the backscattering of electrons, thereby leading to conductivity correction of the form $\sigma \propto \frac{e^2}{\pi h} ln \frac{l_\Phi}{l}$[5]. Applying a

magnetic field breaks the time-reversal symmetry and suppress the WAL. Moreover, suppression of WAL can also be expected for electrons experiencing magnetic scattering due to time-reversal symmetry breaking. As the Berry phase is altered from π to 0 or 2π, a competing WL contribution shows up toward a large-mass limit of Dirac fermion, where WL effect dominates the transport[5]. The weight of WL effect can be quantified by $\Delta/2E_{\mathrm{F}}$, where Δ is the size of exchange gap and E_{F} is the Fermi energy[6].

Our MC data can be fitted to the competing WAL and WL model, using[6]

$$\Delta\sigma_{\mathrm{xx}} = \sigma_{\mathrm{xx}}(B) - \sigma_{\mathrm{xx}}(0) = \sum_{\mathrm{i}=0,1} \frac{\alpha_{\mathrm{i}} e^2}{\pi h}\left[\Psi\left(\frac{\hbar}{4el_{\phi\mathrm{i}}^2 B} + \frac{1}{2}\right) - ln\left(\frac{\hbar}{4el_{\phi\mathrm{i}}^2 B}\right)\right] + \mathrm{c}B^2 \quad (5)$$

, where Ψ is the digamma function and $l_{\phi\mathrm{i}}$ is the phase coherence length. The first two terms represent WL and WAL, respectively, characterized by the prefactors $\alpha_0 > 0$ for WL and $\alpha_1 < 0$ for WAL. The B^2 term results from Lorentz deflection of electrons and c is related to the electron mobility[7]. Comparing the fitting results shown in Supplementary Fig. 7(b) and (c), the MC of Bi_2Se_3(7)/Al_2O_3 can be well fitted to a single WAL effect plus B^2 dependence (WAL+B^2), while that of Bi_2Se_3(7)/YIG cannot be fitted with the same procedure. Instead, a WL contribution should be added (WAL+WL+B^2) to fit the data (Supplementary Fig. 7(d)). Supplementary Table 1 shows the fitting results. There are two points to be noted. Firstly, the fitting for Bi_2Se_3(7)/YIG show larger weight of WL than that of WAL ($|\alpha_0| > |\alpha_1|$) in MC, implying a sizable modification of the Berry phase from π. Secondly, $l_{\phi0}$ and $l_{\phi1}$ of Bi_2Se_3(7)/YIG are substantially smaller than $l_{\phi1}$ of Bi_2Se_3(7)/Al_2O_3. The decreased $l_{\phi0}$ and $l_{\phi1}$ can be viewed as a consequence of magnetic scattering of electrons at the interface.

Our analyses strongly suggest the existence of MPE in our Bi_2Se_3/YIG. Similar

negative MR feature has been readily observed in Bi_2Se_3/EuS[8], where the authors attributed the observations to MPE in Bi_2Se_3/EuS.

Supplementary References

1 d'Allivy Kelly, O. *et al.* Inverse spin Hall effect in nanometer-thick yttrium iron garnet/Pt system. *Appl. Phys. Lett.* **103**, 082408 (2013).

2 Gallagher, J. C. *et al.* Exceptionally high magnetization of stoichiometric $Y_3Fe_5O_{12}$ epitaxial films grown on $Gd_3Ga_5O_{12}$. *Appl. Phys. Lett.* **109**, 072401 (2016).

3 Chang, H. *et al.* Nanometer-Thick Yttrium Iron Garnet Films With Extremely Low Damping. *IEEE Magn. Lett.* **5**, 6700104 (2014).

4 Smit, J. & Beljers, H. G. Ferromagnetic resonance absorption in $BaFe_{12}O_{19}$. *Philips Res. Repts.* **10**, 113-130 (1955).

5 Lu, H. Z. & Shen, S. Q. Weak localization and weak anti-localization in topological insulators. *Proc. of SPIE* **9167**, 91672E (2014).

6 Lu, H. Z., Shi, J. & Shen, S. Q. Competition between weak localization and antilocalization in topological surface states. *Phys. Rev. Lett.* **107**, 076801 (2011).

7 He, H. T. *et al.* Impurity Effect on Weak Antilocalization in the Topological Insulator Bi_2Te_3. *Phys. Rev. Lett.* **106**, 166805 (2011).

8 Yang, Q. I. *et al.* Emerging weak localization effects on a topological insulator–insulating ferromagnet Bi_2Se_3-EuS interface. *Phys. Rev. B* **88**, 081407(R) (2013).
